# Application of Magnetic Resonance DTI Technique in Evaluating the Effect of Postoperative Exercise Rehabilitation

**DOI:** 10.1155/2022/2385699

**Published:** 2022-03-21

**Authors:** Jinping Sheng, Rui Jiang, Feizhou Du, Yang Wang, Xiao Zhang

**Affiliations:** Department of Radiology, The General Hospital of Western Theater Command, Chengdu, Sichuan 610083, China

## Abstract

Magnetic resonance diffusion tensor imaging (DTI) is a new kind of magnetic resonance imaging technology. Its imaging principle is to distinguish different pathological tissues according to the movement of water molecules, which is higher than regular magnetic resonance diffusion-weighted imaging. Magnetic resonance diffusion tensor imaging has exact utility price in medical analysis and sickness evaluation. However, there are few researches on the utility of diffusion tensor imaging in the rehabilitation comparison of patients. This paper explores the utility of magnetic resonance DTI science in evaluating the impact of postoperative patients' exercising rehabilitation. Taking stroke patients as an example, through giving patients rehabilitation training method, using magnetic resonance DTI technology, the motor function rehabilitation of patients was evaluated, and FA changes of the affected side and healthy side and Fugl–Meyer score of two groups of patients before and after rehabilitation were observed. The software outcomes exhibit that, in the contrast of rehabilitation therapy impact of motor feature in sufferers with cerebral infarction, the use of magnetic resonance DTI technological know-how gives a foundation for clinicians to deeply apprehend the CST involvement of patients, which helps to scientifically evaluate the effect and quality of limb motor rehabilitation training of patients and provides a basis for disease treatment.

## 1. Introduction

Nuclear magnetic resonance diffusion tensor imaging (DTI) is a new magnetic resonance technological know-how developed from traditional MRI scanning in the current years. It can quantitatively analyse the diffusion movement of water molecules in tissue in three-D space and can be imaged in accordance with the anisotropic traits of the diffusion movement of water molecules in tissue [1]. Diffusion tensor tractography (DTT) is a technique to gain the photograph of white remember tracts of the intelligence via processing the statistics of white be counted fibres gathered by DTI with distinct laptop software. DTI and DTT blended with traditional MRI and DWI can accurately detect the adjustments of intelligence lesions, so as to decide the relationship between Genius lesions and white be counted fibre tracts and the harm of white be counted fibre tracts[2]. DTI has a proper impact on the distinction between white count number and gray count in the brain. It can produce an accurate imaging impact on the path of white be counted fibre tracts and recognise the compression displacement, infiltration, and destruction of white rely fibre tractsbrought about via lesions [3]. It can grant extra facts for the analysis and differential analysis of lesions and supply extratheoretical groundwork for the improvement of individualized surgical layout and postoperative follow-up.

Cerebral infarction is one of the common clinical cerebrovascular diseases, which has the characteristics of high mortality and disability. According to the survey, 75% of cerebral infarction patients in China will be accompanied by different degrees of limb dysfunction, of which 10% will be accompanied by severe disability, which directly affects the prognosis and quality of life of patients. Limb function rehabilitation is an effective way to improve motor dysfunction after cerebral infarction [4]. Magnetic resonance diffusion tensor imaging is a new magnetic resonance imaging technique, which can exhibit the adjustments of white depend fibre tracts in vivo, which is convenient for the clinical understanding of the involvement of the corticospinal tract and determining the degree of brain lesions [5]. In this paper, we analysed the application of magnetic resonance DTI in evaluating the degree of CST lesions and the effect of rehabilitation treatment before and after rehabilitation treatment of cerebral infarction, in order to discover the medical utility price of magnetic resonance DWI [6].

This paper frequently describes the content material of the paper from five parts, and the particular association of every chapter is summarized as follows: Section 1 is the introduction, which on the whole introduces the particular historical past and lookup magnitude of the paper, analyses the cutting-edge scenario of magnetic resonance DTI science in the assessment of postoperative patients' motor rehabilitation effect, and summarizes the major lookup content material and paper shape arrangement.  Section 2 discusses the associated work.  Section 3 introduces the fundamental precept and quantitative parameters of magnetic resonance DTI.  Section 4 analyses the software of magnetic resonance DTI technological know-how in evaluating the impact of postoperative workout rehabilitation.  Section 5 summarizes the potentialities of this paper.

## 2. Related Work

Conventional MRI can determine the location and scope of intracranial tumours, which has been consensus, but it still has great limitations for the qualitative and staging of intracranial tumours. In current years, home and overseas students have observed that DTI performs an extraordinarily necessary function in the qualitative identification of intracranial tumours, the differentiation of specific factors of tumours, and the identification of tumour infiltration and Genius enema. Magnetic resonance DTI technological know-how has regularly turned out to be a lookup hotspot of pupils at domestic and abroad. The utility of magnetic resonance DTI science has been extensively involved and developed in the latest years. The utility of magnetic resonance DTI science in the impact of exercising rehabilitation is as follows.

It was once determined that the FA price of the tumour aspect was once extensively decreased compared to that of the healthful aspect [7]. The reason may be the invasion of tumour cells, which destroyed the space between nerve fibres and axons. However, the infiltration of water molecules and the necrosis in the tumour lead to the accelerated diffusion of water molecules in all directions, which reduces the diploma of anisotropy. Combined with DTI and 1H-MRS, we found that the FA value of the peritumoral tissue to the tumour core tissue gradually decreased, which reflected the continuous decrease of the degree of tissue structure, consistent with the changes of NAA, and the changes of both reflected the decrease of the structure of neurons and the integrity of fibre bundles [8]. In the enema location around the tumour, the MD price was once appreciably greater than that of everyday intelligence tissue due to the enlargement of extracellular water molecules; the MD price of the tumour core location may additionally be between the enema region around the tumour and the tumour core place due to telephone proliferation, necrosis, and cystic degeneration. The enhanced tumour margin on MRI may be due to more proliferative tumour cells, resulting in increased cell density and lower MD value [9].

Relevant scholars believe that DTI can distinguish the main body of glioma from tumour infiltrating enema area, with a sensitivity of 98% and a specificity of 81%. They can be divided into the following types according to the invasion of white matter fibre tracts displayed by DTI: progressive type: the position and shape of white matter fibre on the diseased side are changed compared with the normal white matter fibre on the opposite side [10]. The FA value does not change significantly when the compression is light but increases when the compression is obvious, which is due to the tight arrangement of fibres caused by obvious compression. Infiltration type: the location and morphology of white matter fibres on the diseased side were abnormal compared with the normal white matter fibres on the opposite side. Destructive type: the FA value decreased significantly in the tumour surrounding enema and tumour growth site, and the white matter fibres in the tumour site were occupied by the tumour tissue and disappeared [11]. According to the results of DTI white matter tractography in patients with intracranial tumors, tumors can be divided into four types: displaced, enema, infiltrating, and destructive [12]. The three-dimensional reconstruction image technology of the corticospinal tract using DTI imaging can accurately guide the surgical resection of intracranial tumors.

Conventional MRI can determine the location and scope of intracranial tumours, which has been a consensus, but it still has great limitations for the qualitative and staging of intracranial tumours. Price et al. found that the FA value of the tumour side was significantly lower than that of the contralateral side. The analysis may be due to the invasion of tumour cells, which destroyed the space between nerve fibres and axons. The infiltration of water molecules and the necrosis of the tumour lead to the accelerated diffusion of water molecules in all directions, which reduces the degree of anisotropy. Goebel and other scholars combined DTI and 1H-MRS to study glioma and found that the FA value of peritumoural tissue to tumor core tissue gradually decreased, which reflected the continuous decrease of tissue structure, consistent with the change of NAA, and the changes of both reflected the decrease of neuron structure and fibre bundle integrity. Goebel and other scholars also conducted a DTI study on a total of 23 patients with gliomas. It was found that the fibre tracts around low-grade gliomas were less invaded, whilst most of the fibre tracts around high-grade gliomas were invaded.

## 3. Magnetic Resonance Diffusion Imaging

### 3.1. The Basic Principle of DTI

Dispersion is a form of random, colliding, and surpassing action of dependent molecules in nature, particularly Brownian motion. The dispersion of free molecules in pure liquid is isotropic; that is, the dispersion movement in all instructions is uniform. In human intelligence tissue, it is affected via the traits of tissue cells and the inner shape of cells. In the tissue shape with constant arrangement, mainly in the nerve conduction tract, such as the corticospinal tract, the diffusion of water molecules in a number of instructions is different. Generally, it is extrainclined to diffuse alongside the course of the nerve fibre bundle, however, hardly ever alongside the course perpendicular to the nerve fibre bundle [13]. Diffusion tensor imaging is based on this principle, by applying diffusion sensitive gradients in multiple directions and calculating the characteristic vector values of the main diffusion directions in each voxel of brain tissue; the FA map, colour tensor map, and white matter fibre bundle map of white matter structure are obtained by postprocessing. The signal intensity in FA image represents the degree of anisotropy of brain tissue diffusion. It can directly show the shape of white matter fibre tracts but cannot show the direction of its course [14]. The colour tensor map is generated by postprocessing the direction and size of the largest eigenvector value in each voxel with three different colours: green, red, and blue. Red represents the direction of left and right fibre bundles, green represents the direction of front and back fibre bundles, and blue represents the direction of head and tail fibre bundles; it additionally confirmed the path of the white be counted fibre tracts. On the groundwork of the former two, the map of white count number fibre tracts suggests the location, course, morphology of the white be counted fibre tracts in the Genius and the anatomical relationship with the surrounding tissue structure. Zhang Weeding and different researchers discovered that the white depend fibre tracts (mainly corticospinal tracts) passing via the posterior limb of the inside pill and the white remember fibre tracts in the knee and compression phase of the visceral corpus callosum have been without a doubt displayed by using diffusion tensor imaging. This is in good agreement with the known anatomical knowledge, which indicates that this new imaging technique has objectivity and clinical practicability. In this study, the patients in the experimental group improved the operation plan according to the anatomical position relationship between glioma and corticospinal tract provided by DTI before operation and achieved good results in protecting the fibre bundle during operation, which was statistically different from that in the control group [15]. It also showed that the fibre bundle information provided by DTI was in good consistency with the known fibre bundle anatomy and the physical fibre bundle in the human body; it can provide reliable information for resection of glioma and protection of important white matter fibre tracts during operation.

### 3.2. Quantization Parameters of DTI

**Figure 1:**
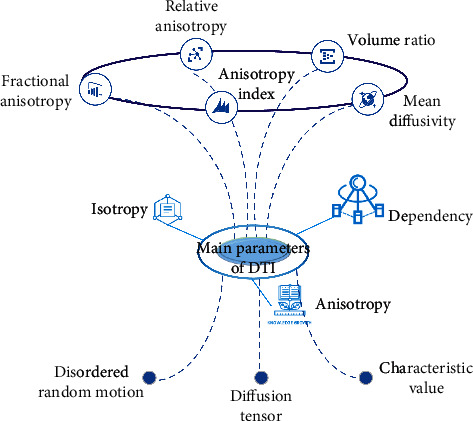
Schematic format of essential parameters of DTI.

DTI is a new magnetic resonance imaging method based totally on traditional MRI and DWI. The random movement of molecules and other microparticles from high concentration to low concentration is called dispersion, which is one of the ways of material movement. In the homogeneous liquid outside the human body, the water molecules move randomly, and the probability of moving in all directions is the same, which is called dispersion isotropy. In the human body, water molecules are affected by different tissues, structures, and other factors in the process of movement, resulting in different diffusion velocity in three-dimensional space. Diffusion in one direction is more limited than that in the other direction, which has a robust route dependence, which is known as anisotropy [16]. DTI is the use of human tissue water molecules dispersion anisotropic traits of magnetic resonance diffusion imaging; DTI according to the anisotropic characteristics of water molecules dispersion movement in nerve fibres can reflect its unique tissue anatomical structure and can provide valuable information about the microstructure and pathological state of the tissue. The main parameters of DTI are FA (fractional anisotropy), RA (relative anisotropy), R (volume ratio), MD (mean dispersion), VAI (anisotropy index), and so on. The schematic format of essential parameters of DTI is proven in Figure 1.

The action of water molecules in homogeneous media is random and disordered; that is, the chance of transferring in all instructions is the same; that is, it has isotropy. In human tissues, the motion of water molecules is affected by way of the shape of tissues and cells, and the degree of dispersion is different in all directions, which is direction-dependent, that is, anisotropic. In homogeneous media, the free motion of water molecules can be isotropic; that is, the dispersion intensity in all directions is the same. The dispersion tensor D is described as a sphere, and the eigenvalues along the three principal coordinates of MR are *λ*_1_=*λ*_2_=*λ*_3_.

**Figure 2:**
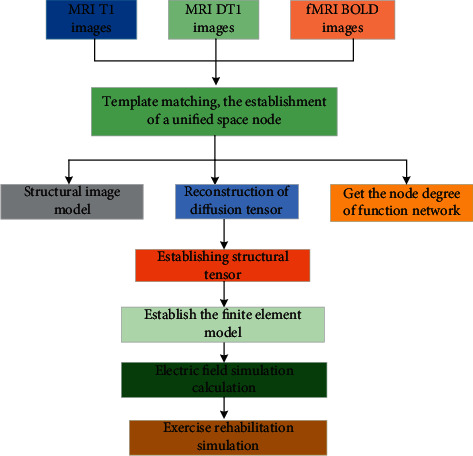
Exercise rehabilitation model based on DTI.

In the white matter, due to the blockading of myelin sheath, the diffusion of water molecules is restrained in the identical route as the fibre, which has a high anisotropy. At this time, the diffusion tensor can be expressed as an ellipsoid with its eigenvalue *λ*_1_=*λ*_2_=*λ*_3_. The direction corresponding to the maximum eigenvalue is parallel to the fibre bundle passing through the voxel. The exercise rehabilitation model based on DTI is shown in Figure 2.

The common fee of diffusion amplitude in every course of MR voxel represents the dimension or diploma of diffusion of water molecules in a voxel. In many instances, the used index is the apparent diffusion coefficient (ADC), which represents the change in the diffusion of water molecules per unit time. The unit is mm^2^/s. The larger the cost is, the more improved the diffusion potential of water molecules is. The most normally used parameter for analysing anisotropy is the ratio of the anisotropic phase of the dispersion to the whole price of the dispersion tensor, which displays the share of the anisotropic element in the entire dispersion tensor. The price is between zero and 1. Zero represents the most isotropic dispersion, such as the dispersion of water molecules in a totally homogeneous medium, and 1 represents the hypothetical most anisotropic dispersion. FA fee in white be countedused to be positively correlated with myelin integrity, fibre density, and parallelism [17]. Relative anisotropy (RA) and extent ratio (VR) are the percentage of anisotropic and isotropic components. VR is equal to the ratio of the quantity of the ellipsoid to the extent of the sphere whose radius is the common diffusivity. The fee of RA is comparable to that of FA. The nearer the RA to 1, the greater the anisotropy of water molecules. The nearer the VR to 1, the greater the isotropy of the dispersion of water molecules.

## 4. Application Value of Diffusion Tensor Imaging

Traditional brain tumour surgery focuses on relieving the space-occupying effect of brain tumour. In the operation, it is emphasized to remove the tumour as far as possible on the premise of protecting the cerebral cortex, nerves, and important intracranial vessels. However, with the development and establishment of the concept of microinvasion in recent years, the development of microinvasive neurosurgery for brain tumours has paid more and more attention to the protection of patients' neurological function and the quality of life after operation. The last intention is to enhance the exceptional lifestyles and survival time of patients. The emergence of DTI technology provides the possibility for this purpose. It is a major development of MRI technology in recent years, and it is the only way to display the white matter fibre tracts in vivo. By evaluating the movement state of water molecules in the microenvironment, it can not only infer the degree of infiltration and destruction of white matter fibre tracts in brain tumours from the microscopic perspective but also show the extent of brain tumour invasion and its relationship with important white matter tracts. The key to improving the quality of life of patients after operation is to protect the important cerebral cortex and white matter fibre tracts as much as possible. The emergence of functional magnetic resonance (fMRI) helps neurosurgeons to solve the problem of protecting not only the important cerebral cortex but also the important cerebral white matter fibre tracts, mainly the corticospinal tract, corpus callosum, optic radiation, and so on; the safety of acoustic radiation and different fibre tracts has been perplexing neurosurgeons, which leads to two kinds of adverse mentality: (1) in order to completely remove the tumour tissue as much as possible, the operation is too deep, and the uninvolved pyramidal tract is removed. The postoperative limb dysfunction was aggravated. (2) In order to protect the preoperative limb function, the operation is too conservative. The rate of total tumour resection decreased, and the emergence of DT technology solves this contradiction, because of the white matter fibre bundle. The diffusion direction of water molecules is limited by the fibre sheath of white matter, which has high anisotropy and tends to be the direction of the vessel fibre bundle. Therefore, different nerve conduction and fibre projection directions can be clearly distinguished, especially the corpus corporis bundle and the corpus callosum fibre bundle. Fourth, the anisotropy of the fibre bundle is obvious and the strip structure is high signal. According to the FA colour map and DTT map, we can accurately determine the shape structure, course of the corresponding white matter fibre, and its spatial relationship with adjacent tumours before operation, which is helpful to the preoperative evaluation and operation planning design of brain tumour surgery. In this study, the experimental group patients were treated according to the fibre bundle image information provided by DITI technology before operation [18]. By improving the surgical plan as much as possible, and protecting the corticospinal tract in a targeted manner, the aggravation of limb dysfunction in patients after surgery will be significantly reduced.

## 5. Experimental Design

In this study, we used DTI to monitor the parameters of the focal area and symmetrical normal brain area in patients with newly diagnosed cerebral infarction in acute phase, subacute phase, and chronic phase, mainly including average diffusion coefficient (Drag), volume ratio anisotropy (VRA) and fractional anisotropy, and exponential attenuation. At the same time, the sufferers have been divided into rehabilitation team and nonrehabilitation team in accordance with MBI score, and the parameters of lesion place and contralateral unhurt area, rehabilitation crew, and nonrehabilitation crew have been in contrast at unique time periods, so as to comprehensively analyse the correlation between the above parameters and rehabilitation diploma of sufferers with cerebral infarction with the aid of magnetic resonance diffusion tensor imaging (DTI), to inform the medical improvement of extra optimized rehabilitation treatment.

### 5.1. General Information

Patients with persistent cerebral infarction admitted to our medical institution from September 2019 to September 2020 have been chosen as the lookup objects, which have been tested by CT or MRI examination and met the diagnostic standards formulated in 2010 Chinese guiding principle for the analysis and cure of acute ischemic stroke. Inclusive criteria include the following: (1) admission for the first time; (2) all lesions being unilateral and involving white matter; (3) with different degrees of motor dysfunction; (4) the clinical data being complete; (5) absence of any other intracranial lesions; (6) providing informed consent. Exclusion criteria include the following: (1) contraindications of MRI examination; (2) combined with mental illness; (3) hearing or language impairment; (4) having serious physical diseases before enrolment, which affected the limb motor rehabilitation treatment; (5) the treatment compliance being poor. The age ranged from 39 to 72 years, with an average of 51.26 ± 2.63 years. The duration of cerebral infarction ranged from 19 days to 76 days (36.31 ± 5.18 d).

### 5.2. Rehabilitation Training Method

After the vital signs were stable, all patients received hyperbaric oxygen, brain nerve nutrition, acupuncture and massage, passive limb training, and other rehabilitation treatment programs. Hyperbaric oxygen treatment pressure, pressure time, decompression, and course of treatment were formulated according to clinical standards. Brain nutrition includes the application of brain neurotrophic factor, glial neurotrophic factor, brain protein hydrolysate, traditional Chinese medicine decoction, and so on. For acupuncture, special metal needles are used to penetrate the relevant blood levels, such as Wanigan, Qu chi, Taichung, and USANi, so as to dredge channels and activate collaterals and regulate qi and blood. Massage uses a variety of techniques to stimulate the blood vessels, muscles, nerves, and other parts of the patient, so as to speed up the metabolism and help them recover their functions as soon as possible; passive and active limb training include keeping good posture, elbow extension training, and wrist dorsiflexion training; the patients should be guided to exercise autonomously as far as possible, the affected limb should be encouraged to do lifting activities, and the walking training should be strengthened to adjust the flexion movement of the knee joint of the affected limb; language rehabilitation training includes starting with words and single sentences, then strengthening the practice of complex sentences, and encouraging more communication with family members. The above rehabilitation treatment methods were carried out according to clinical standards under the guidance of doctors and nurses.

### 5.3. Inspection Method

HDX twin speed 3.0TMR scanner produced by GE company of America is used as the inspection instrument, and standard thread end ring is used. The scanning sequences were T1WI, T2WI, FLAIR, and DTI. DTI scanning technology is single-shot spin-echo plane echo imaging (EPI); scanning parameters are as follows: TR/TE is 8000 ms/96 ms, weighted coefficient is *b* = 0, *B* = 1000 s/mm^−2^, scanning matrix is 160 × 160, and field of vision is 22 × 22 cm. The scanning range was from skull base to skull top. The scanned image was transmitted to the workstation for processing. FA reconstruction was performed to determine the region of interest with an area of 15 mm^2^, and FA values of the region and the contralateral normal region were obtained. The relationship between the lesion and CST was observed and divided into three grades: grade 1: the morphology of CST was complete and the trend was normal; grade 2: CST has pressure and trend change; grade 3: CST interrupt.

In this study, we used the signal HDE 1.5T MRI scanner of GE company to collect the images. The patient took the head first, in a supine position. During the scanning, the patient was asked to rest and relax his limbs. The scanning sequence included axial t1flair (TR 1846 ms, the 28.6 ms, matrix 288 × 192, field of vision 240 mm × 240 mm, and 7 mm in thickness); axial T2WI (TR 5000 ms, TE 132 ms, matrix 288 × 288, visual field 240 mm × 240  mm, and thickness 7 mm); axial t2flair (TR 8400 ms, TE 148 ms, matrix 288 × 288, visual field 240 mm × 240  mm, and thickness 7 mm); axial DWI (TR 8850 ms, TE 108 ms, matrix 128 × 128, field of vision 240 mm × 240 mm, and 7 m in thickness); sagittal 2WI (TR 2500 ms, TE 88.1 ms, matrix 96 × 128, field of vision 240 mm × 240 mm, and thickness 5 mm); axial DTI (TR 8000 ms, TE 14 88.1 ms, matrix 96 × 128, field of vision 240 mm × 240 mm, and thickness 4 mm).

### 5.4. Observation Index

(1) The objective is to check out the relationship between pathological adjustments and CST. (2) According to the relationship between sickness and CST, sufferers in team A have been grade 2, and sufferers in team B have been grade 3. (3) Two hours earlier than every MRI examination, the motor feature recuperation of the sufferers used to be evaluated by using the easy Fugl–Meyer motor characteristic scale, together with top limb function, finger function, decrease limb function, and so on. The whole rating used to be zero to a hundred. The more the decrease in the score, the more serious the motor dysfunction. The correlation between FA and Fugl-Meyer scores before and after rehabilitation training has been discussed.

### 5.5. Statistical Methods

The collected data were sent to the workstation, FA, ADC, *DC*_avg_, and VRA maps were reconstructed, and the infarction area of 15 mm^2^in the image was selected as the region of interest (ROI), including the central part and surrounding part of the infarction. FA value, *DC*_avg_value, VRA value, and *E*_xat_value were measured in the infarction area, respectively. FA value, *DC*_avg_value, VRA value, and *E*_xat_value were measured in the control area. Spss22 software was used to analyse the data. For data ($\bar{x}\pm s$), the difference was statistically significant (*P* < 0.05).

## 6. Results and Analysis

### 6.1. Changes of FA in Different Parts and Periods before and after Rehabilitation

**Figure 3:**
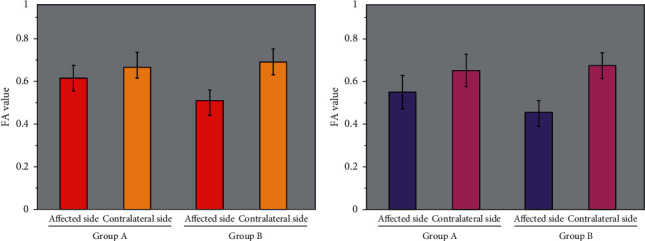
Changes of FA value in affected side and healthy side of cerebral peduncle before and after rehabilitation. (a) Before recovery. (b) After recovery.

15 cases of grade 2 were treated as group A, and 21 cases of grade 3 were treated as group B. The FA value of group A was 0.612 ± 0.039, which was significantly higher than that in group B. Compared with before rehabilitation, the difference was significant (*P* < 0.05). The FA value of group A was 0.556 ± 0.064, which was significantly higher than that in group B (*P* < 0.05), as shown in Figure 3.

15 instances of grade two have been dealt with as team A, and 21 cases of grade three had been dealt with as crew B. The FA cost of crew A (0.612 ± 0.039) was once extensively greater than that in team B. Compared with earlier than rehabilitation, the distinction was once huge (*P* < 0.05). The FA cost of group A (0.556 ± 0.064) was once much larger than that of group B (*P* < 0.05), as proven in Figure 3.

**Figure 4:**
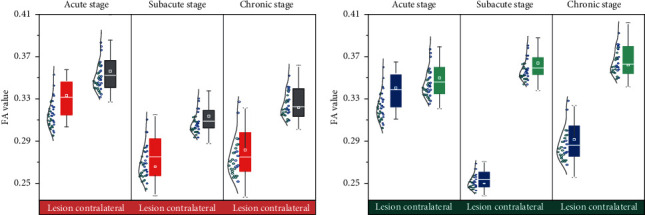
Comparison of FA value between the rehabilitation group and the nonrehabilitation group in unique durations. Comparison of VRA values in two groups under different recovery conditions. (a) Rehabilitation group. (b) Nonrehabilitation group.

The FA fee of ROI in the rehabilitation team and the nonrehabilitation crew used to substantially decrease compared to that in the contralateral facetin the subacute and persistent segment (*P* < 0.05), and the FA fee of the nonrehabilitation team in the subacute section used to substantially decrease compared to that in the rehabilitation crew (*P* < 0.05). In the continual phase, the FA fee of ROI in the rehabilitation team did now not alternate extensively in contrast with that in the subacute phase; however, the distinction was once large than that in the nonrehabilitation team (*P* < 0.01), as proven in Figure 4.

### 6.2. Comparison of VRA Values in Two Groups Under Different Recovery Conditions

**Figure 5:**
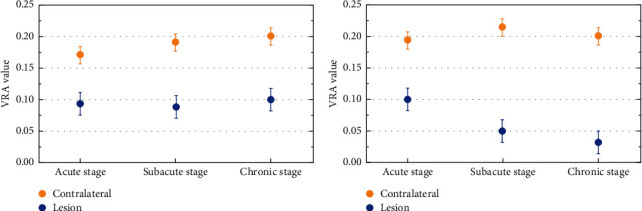
Comparison of VRA value between rehabilitation group and nonrehabilitation group in different periods. (a) Rehabilitation group. (b) Nonrehabilitation group.

In the acute phase, the VRA price of the two agencies used to considerably decrease compared to that of the contralateral ROI (*P* < 0.05). The VRA cost of ROI in the rehabilitation crew was once no longer considerably modified in contrast with that in the acute stage, whilst the VRA cost of ROI in the nonrehabilitation team persevered to decrease, and there used to be a statistical distinction between the two organizations (*P* < 0.05). In the persistent stage, the VRA fee of ROI in the rehabilitation crew used to be nevertheless now not substantially unique from that in the acute and subacute stages. The VRA price of ROI in the nonrehabilitation crew persisted to decrease, and the statistical distinction between the two businesses was once enlarged (*P* < 0.01), as proven in Figure 5. In the acute stage, the VRA of the two groups decreased significantly, and there was no significant difference between the two groups. With the prolongation of the disease duration, the VRA of the two groups differentiated in the subacute stage. The VRA of the nonrehabilitation group continued to decline significantly, whilst that of the rehabilitation group slowed down, and the difference between the two groups was statistically significant (*P* < 0.05). There was no difference in VRA between the rehabilitation group and its acute and subacute stages (*P* > 0.05), but the VRA of the nonrehabilitation group decreased continuously, and the difference between the two groups was enlarged (*P* < 0.01).

### 6.3. The Difference of *DC*_avg_ between Rehabilitation Group and Nonrehabilitation Group

**Figure 6:**
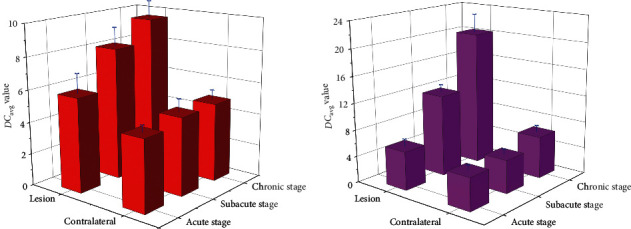
Comparison of *DC*_avg_ value between rehabilitation group and nonrehabilitation group in different periods. Comparison of *E*_xat_ value between rehabilitation group and nonrehabilitation group. (a) Rehabilitation group. (b) Nonrehabilitation group.

The *DC*_avg_ price of the two organizations was once appreciably greater than that of the contralateral ROI in the acute segment (*P* < 0.05); however, there was once no widespread distinction in the ROI between the two businesses in the acute phase. In the acute stage, the *DC*_avg_ fee of the two agencies persisted to increase, and the *DC*_*avg*_ fee of the rehabilitation group used to be drastically greater than that of the rehabilitation team (*P* < 0.05). In the continual phase, the *DC*_avg_ cost of the rehabilitation team accelerated slowly, whilst the *DC*_avg_ cost of the nonrehabilitation team persevered to increase, and the enlargement used to be apparently greater than that of the rehabilitation team (*P* < 0.01), as proven in Figure 6.

### 6.4. Comparison of Exat Value Between Rehabilitation Group and Non-Rehabilitation Group

**Figure 7:**
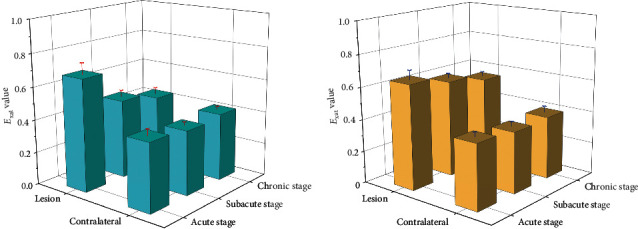
Comparison of *E*_xat_ value between rehabilitation group and nonrehabilitation group in different periods. Comparison of Fugl–Meyer scores before and after rehabilitation. (a) Rehabilitation group. (b) Nonrehabilitation group.

The *E*_xat_ value and contralateral ROI of the rehabilitation group were significantly higher than those of the nonrehabilitation group (*P* < 0.05); after the subacute stage, the *E*_xat_ value of the rehabilitation group decreased significantly, and there was no statistical difference with the contralateral ROI, whilst the *E*_xat_ value of nonrehabilitation group decreased slowly, and there was a statistical difference with the contralateral ROI and the ROI of rehabilitation group; after the disease developed to the chronic stage, the *E*_xat_ value of the rehabilitation group returned to the normal level, whilst the *E*_xat_ value of the ROI of the nonrehabilitation group decreased slowly, and there was a statistical difference between the contralateral ROI and the ROI of the rehabilitation group (*P* < 0.05), as shown in Figure 7.

### 6.5. Comparison of Fugl-Meyer Scores Before and After Rehabilitation

**Figure 8:**
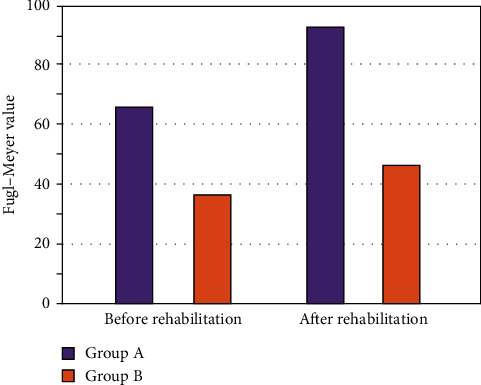
Comparison of Fugl–Meyer score before and after rehabilitation treatment.

Before rehabilitation, the Fugl–Meyer rating of crew A used to be 57.264 ± 5.821, which was once extensively greater than that of crew B. After rehabilitation, the Fugl–Meyer rating of crew A used to be 85.162 ± 9.861, which used to be extensively greater than that in team B, and the variations had been statistically substantial (*P* < 0.05). There was once a terrible correlation between the limit share of CST FA cost and the expanded proportion of Fugl–Meyer rating earlier than and after rehabilitation treatment, as proven in Figure 8.

## 7. Discussion

DTI is a new type of MR technology based on traditional DWI technology. At present, DTI is the only noninvasive method that can effectively track the white matter fibre tracts of patients. In recent years, it is more and more used in the diagnosis, treatment, and follow-up of nervous system-related diseases. There are many characteristic parameters of tissue diffusion commonly used in DTI. This study mainly compares the changes of FA value, *DC*_avg_value, VRA value, and *E*_xat_value of DTI in different periods to explore the evaluation value of DTI in the subacute stage for patients' later rehabilitation [19]. FA refers to the internal characteristics of different tissues. It is often used to measure the integrity, compactness, and parallelism of myelin sheath. It can sensitively reflect whether the fibre microstructure in white matter is damaged. The significant decrease of FA value means that the integrity of cells is damaged or irreversible cell damage occurs. The change direction of VRA value is the same as that of FA value, which can also reflect the anisotropy in tissues. The difference is that FA value can reflect the lower anisotropy in tissues, whilst VRA value can reflect the higher anisotropy in tissues. Therefore, this study used VRA value as a supplementary index of FA value to comprehensively evaluate the structure of cerebral infarction lesions. Drag value represents the diploma of free diffusion of molecules in unit time, which is associated with diffusion velocity; however, it has negative correlation with anisotropy. The large the *DC*_avg_ value, the larger the diploma of diffusion of water molecules in tissue and the freer the water content material in tissue; *E*_xat_ value, additionally recognised as ADC, is the equal to *DC*_avg_ and can additionally mirror the diffusion traits of water molecules in tissues; however, they replicate the contrary course [20, 21].

This confirmed that there used to be no massive distinction in the FA cost of ROI between the rehabilitation crew and the nonrehabilitation team in the acute stage. With the extension of the disease, the FA price of the two corporations was once drastically decreased compared to that of the contralateral crew (*P* < 0.05). At the equal time, the FA price of the nonrehabilitation team used to considerably decrease compared to that of the rehabilitation crew (*P* < 0.05). It can be determined that the FA price diminished much less in subacute stage, and the sufferers recovered higher in later stage. In the detection of VRA value, as a supplementary index of FA value, it displays quicker than FA value. In the acute stage, the VRA fee of the two agencies lowered significantly, and there was once no massive distinction between the two groups. With the extension of the disorder time, the VRA cost of the two organizations differentiated in the subacute stage. The VRA fee of the nonrehabilitation crew persevered to decline significantly, whilst the VRA cost of the rehabilitation crew diminished slowly, and the distinction of VRA between the two companies used to be statistically sizable (*P* < 0.05). In the persistent stage, there used to be no distinction in the VRA between the rehabilitation team and its acute and subacute levels (*P* > 0.05); however, the VRA of the nonrehabilitation crew lowered continuously, and the distinction between the rehabilitation crew and the rehabilitation crew at the identical stage multiplied (*P* < 0.01), FA fee and VRA cost can song the variations inside the tissue, and *DC* avg cost is particularly correlated with the diffusion pace of water molecules. In acute stage, the *DC*avg cost of the two corporations expanded appreciably in contrast with the contralateral facet (*P* < 0.05). In subacute stage, the *DC*_avg_ cost of the two companies confirmed the difference. The *DC*_avg_ fee of the rehabilitation crew elevated slowly, whilst the ROI of the nonrehabilitation crew accelerated continuously. The *DC*_avg_ fee of the two companies confirmed the sizable distinction (*P* < 0.05). The *DC*_avg_ cost of ROI in the rehabilitation crew was once nevertheless extensively special from that in the contralateral side; however the rising vogue was once gentle, whilst that in the nonrehabilitation crew endured to rise, and the distinction between the two businesses grew to become large (*P* < 0.01). The *E*_xat_ cost used to be equal to *DC*_avg_, which ought to additionally mirror the molecular diffusion characteristics. In the acute phase, the *E*_xat_ price of ROI in the two organizations reduced extensively at the identical time, and there used to be a full-size distinction with the contralateral ROI (*P* < 0.05). With the extension of time, the *E*_xat_ cost started to decrease, particularly in the rehabilitation group, the reduce degree in the subacute segment used to be a lot increased than that in the nonrehabilitation team (*P* < 0.05). The ROI of the *E*_xat_ fee for the rehab team is back to normal again, whilst the *E*_xat_cost of ROI in the nonrehabilitation crew was once nonetheless above the regular degree (*P* < 0.05).

The above studies show DTI detection in patients with cerebral infarction in the early stage of a number of abnormal indicators, including FA values in the subacute phase of the difference. The reason for the above changes may be that, in the acute stage of cerebral infarction, neuron cells are first subjected to ischemia and hypoxia, which leads to cell enema and limits the diffusion of water molecules [22]. At this time, the microstructure of nerve fibre bundle has not been significantly damaged, and the structural damage of nerve fibre bundle will take a period of time to gradually show up, which leads to the decrease in FA value [23, 24]. In the subacute stage, although many indicators have abnormal performance, the indicators of ROI in the rehabilitation group tend to be remission or close to the normal level compared with the nonrehabilitation group [25]. DTI can dynamically observe the focus area, accurately judge the phase of the disease, and evaluate the progress of the disease. Especially in subacute cerebral infarction, through the above research, we can find that the different changes of various treatments in subacute cerebral infarction can help to predict the late rehabilitation status of patients and guide the early rehabilitation treatment.

## 8. Conclusion

The utility effects of this paper exhibit that the software of magnetic resonance DTI science in the assessment of rehabilitation cure impact of motor feature in sufferers with cerebral infarction can supply a foundation for clinicians to deeply recognise the CST involvement of patients, help to scientifically evaluate the effect and quality of limb motor rehabilitation training of patients, and provide a basis for disease treatment. As a new technology, the clinical application of DTI is still in its infancy, and there are some problems, such as the distortion of EPI sequence image and the deviation caused by low resolution. At present, there is no “gold standard” for tracing images of nerve fibre tracts. With the development of MRI technology and software, I believe these problems will be solved gradually in the future. However, DTI can supply precious data about the microstructure and pathological kingdom of tissues. It is the solely noninvasive technique to learn about the morphology and shape of white remember fibretracts in human dwelling brain. It has a very broad application prospect in the diagnosis and treatment of central nervous system diseases, especially neurosurgical diseases; the application of DTI in other systems such as heart, kidney, and skeletal muscle system has been reported. With the further development of DTI technology, DTI will be widely used in clinical work.

## Data Availability

The data used to support the findings of this study are available from the authors upon request.
